# Use of HLA peptidomics and whole exome sequencing to identify human immunogenic neo-antigens

**DOI:** 10.18632/oncotarget.6960

**Published:** 2016-01-20

**Authors:** Shelly Kalaora, Eilon Barnea, Efrat Merhavi-Shoham, Nouar Qutob, Jamie K. Teer, Nilly Shimony, Jacob Schachter, Steven A. Rosenberg, Michal J. Besser, Arie Admon, Yardena Samuels

**Affiliations:** ^1^ Department of Molecular Cell Biology, Weizmann Institute of Science, Rehovot, Israel; ^2^ Department of Biology, Technion, Haifa, Israel; ^3^ The Ella Lemelbaum Institute for Melanoma, Chaim Sheba Medical Center, Tel Hashomer, Israel; ^4^ Department of Biostatistics and Bioinformatics, H. Lee Moffitt Cancer Center and Research Institute, Tampa, FL, USA; ^5^ National Cancer Institute, NIH, MD, USA; ^6^ Department of Clinical Microbiology and Immunology, Sackler School of Medicine, Tel Aviv University, Tel Aviv, Israel

**Keywords:** HLA, TILs

## Abstract

The antigenicity of cells is demarcated by the peptides bound by their Human Leucocyte Antigen (HLA) molecules. Through this antigen presentation, T cell specificity response is controlled. As a fraction of the expressed mutated peptides is presented on the HLA, these neo-epitopes could be immunogenic. Such neoantigens have recently been identified through screening for predicted mutated peptides, using synthetic peptides or ones expressed from minigenes, combined with screening of patient tumor-infiltrating lymphocytes (TILs). Here we present a time and cost-effective method that combines whole-exome sequencing analysis with HLA peptidome mass spectrometry, to identify neo-antigens in a melanoma patient. Of the 1,019 amino acid changes identified through exome sequencing, two were confirmed by mass spectrometry to be presented by the cells. We then synthesized peptides and evaluated the two mutated neo-antigens for reactivity with autologous bulk TILs, and found that one yielded mutant-specific T-cell response. Our results demonstrate that this method can be used for immune response prediction and promise to provide an alternative approach for identifying immunogenic neo-epitopes in cancer.

## INTRODUCTION

Antigen presentation through the binding of peptides to cellular HLA molecules modulates T cell response specificity, and thus determines cell antigenicity. CD8+ cytotoxic T lymphocytes (CTLs) have the potential to recognize tumor cells via specific peptide-HLA binding, leading to tumor regression following immunotherapy [1]. One class of antigens promoting this recognition is the tumor-associated self-antigens. However, neo-epitopes, that arise as a consequence of somatic mutations in the tumor, are another antigen subgroup that induces anti-tumor CTL responses [2, 3].

The main screening method currently used to identify neo-antigens relies on identification of tumor somatic mutations by whole-exome sequencing, followed by *in silico* analysis of candidate mutated peptides, to calculate their theoretical affinity for the patient's HLA complex [4]. Synthetic peptides of the selected sequences are then pulsed on antigen presenting cells [2, 5], or loaded on tetramers [6], to evaluate their immunogenicity when introduced to autologous T- cells. The success of this method crucially depends on the accuracy of peptide-MHC binding prediction algorithms, which are often inadequate, and produce large numbers of candidate mutant peptides. Another method, that eliminates the need to predict peptide-MHC binding, is the use of tandem minigenes encoding each expressed cancer-specific mutation. These minigenes are expressed in the patients' autologous antigen presenting cells and then tested for reactivity with the patients' T cells [7, 8]. Subsequent validation of both the abovementioned neo-antigen screening methods is labor-intensive and time-consuming.

The collection of HLA class I (HLA-I) bound peptides expressed by a particular cell, otherwise known as the cell's ‘HLA peptidome’, reflects the set of proteins found in that cell. The HLA peptidome serves as a unique immunological signature that can be selectively recognized by CTLs, potentially leading to cell lysis. Recent advances in mass spectrometry make it possible to analyze the HLA peptidome in great detail [9]. Here, we report the first analysis of the mutated HLA-I peptidome of human melanoma tumor cells, using a novel strategy, that combines whole-exome sequencing and mass spectrometry analysis. Using this method, we experimentally identified mutated peptides that are actually processed and presented by the tumor HLA molecules, and proceed to assess only those empirically identified candidates for testing immunogenicity.

## RESULTS

### Identification of melanoma neo-antigens

To identify mutated antigens presented on human melanoma tumor cells, we re-sequenced the whole genomes of matched normal and metastatic tumor DNAs from a melanoma patient and identified their somatic mutations (Supplementary Table 1).

In parallel to our whole exome analysis, we performed immunoaffinity purification of the HLA molecules from the same patient's melanoma cells, followed by capillary chromatography and tandem mass spectrometric analysis of HLA peptides. The mass spectrometry spectra were analyzed using MaxQuant [10] software tool and queried against the human proteome dataset (Uniprot) to which we manually added the amino acid changes corresponding to the mutations identified by our whole-genome sequencing.

**Figure 1:**
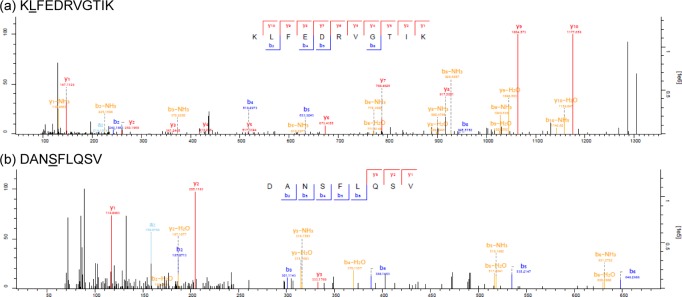
Tandem mass spectra of endogenous mutant peptides identified in 12T **a**. KLFEDRVGTIK **b**. DANSFLQSV. Mutation sites are indicated with the amino acid underlined.

Among the 4,958 peptides detected by mass spectrometry, we identified two patient-derived mutated peptides (Figure 1). The first peptide is a nonamer derived from the P677S alteration in Mediator of RNA polymerase II transcription subunit 15 (MED15) and the second peptide is an elevenmer derived from the S123L alteration in Tumor Protein D52-Like 2 (TPD52L2) protein. Peptide identification accuracy was validated by comparison of endogenous peptide spectra to synthetic peptide spectra (Supplementary Figure 1). Both wild-type and mutant transcripts corresponding to the peptides were expressed in the 12T tumor cell line (Supplementary Figure 2 and Supplementary Table 2).

### Immunogenicity evaluation of the identified neo-antigens

**Figure 2:**
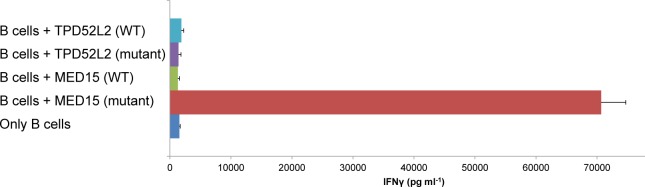
Response of the TIL to candidate neo-antigens identified from its autologous tumor IFN-γ release measured after overnight co-culture of the TIL with autologous EBV transformed B cells that were pulsed with 1μM of the mutated or wild-type peptides. Error bars represent standard deviation of triplicates.

To evaluate the peptides' ability to elicit an immune response we pulsed synthetic MED15 and TPD52L2 mutant peptides and their wild-type version, on Epstein bar virus (EBV)-transformed B cells established from the patients' peripheral blood mononuclear cells (PBMCs) and co-cultured these cells with the autologous TILs. As seen in Figure 2, the mutated MED15 peptide stimulated interferon-γ (IFN-γ) release from the TILs, whereas the mutant TPD52L2 peptide did not show any response.

**Figure 3:**
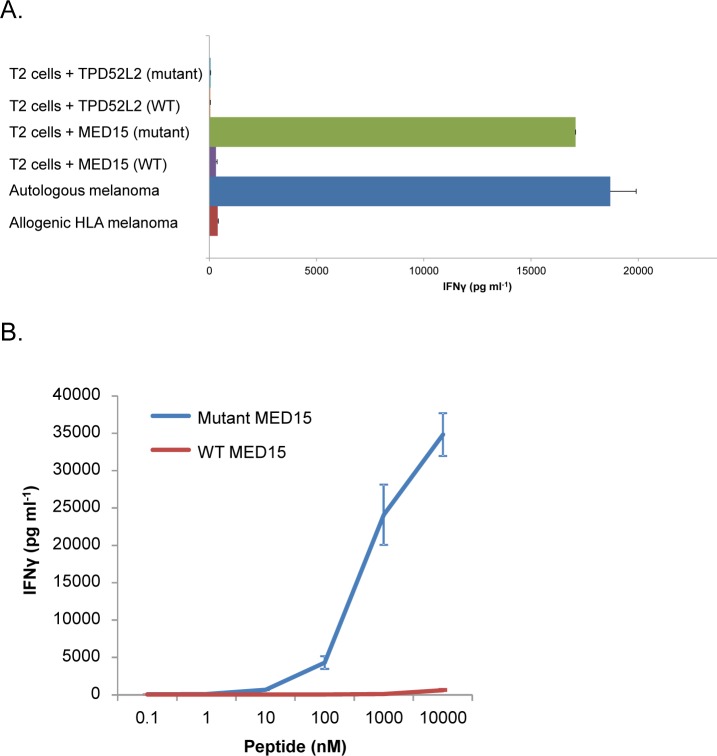
Response of the TILs to MED15 and TPD52L2 mutant and wild-type peptides in the context of HLA-B*51 **A**. IFN-γ release measured after overnight co-culture of the TILs with T2 cells that were pulsed with 1μM of the mutated or wild-type peptides or with the autologous and allogenic melanoma cells. **B**. IFN-γ release measured after overnight co-culture of 12T TILs with T2 cells that were pulsed with tittered concentrations of the MED15 mutated and wild-type peptides. Error bars represent standard deviation of triplicates.

To determine the HLA allele to which the mutant MED15 peptide binds, we pulsed it on cells of the T2 line, which is B*51 positive [11], then cultured the cells with the patient's TILs. Remarkably, the IFN-γ released following pulsing these cells with mutant MED15 peptide was comparable to that measured after TILs stimulation by the patient's autologous tumor cells (17,000 pg ml^−1^ compared to 18,700 pg ml^−1^ with the autologous melanoma), indicating that the peptide binds the B*51 allele. Importantly, when cells of the T2 line were pulsed with the wild-type version of the MED15 peptide, a substantially weaker response was detected (300 pg ml^−1^ in the experiment detailed in Figure 3A).

Peptide titration assays further confirmed that the patient's TILs were highly reactive with T2 cells pulsed with the mutated MED15 peptide, which was recognized at a minimum concentration of 10 nM (Figure 3B). Meanwhile, a response to the MED15 wild-type peptide was only observed when T2 cells were pulsed with peptide concentrations two orders of magnitude higher. Mutant MED15 peptide therefore appears to represent a naturally processed epitope recognized by the patient's TILs (Figure 3B).

**Figure 4:**
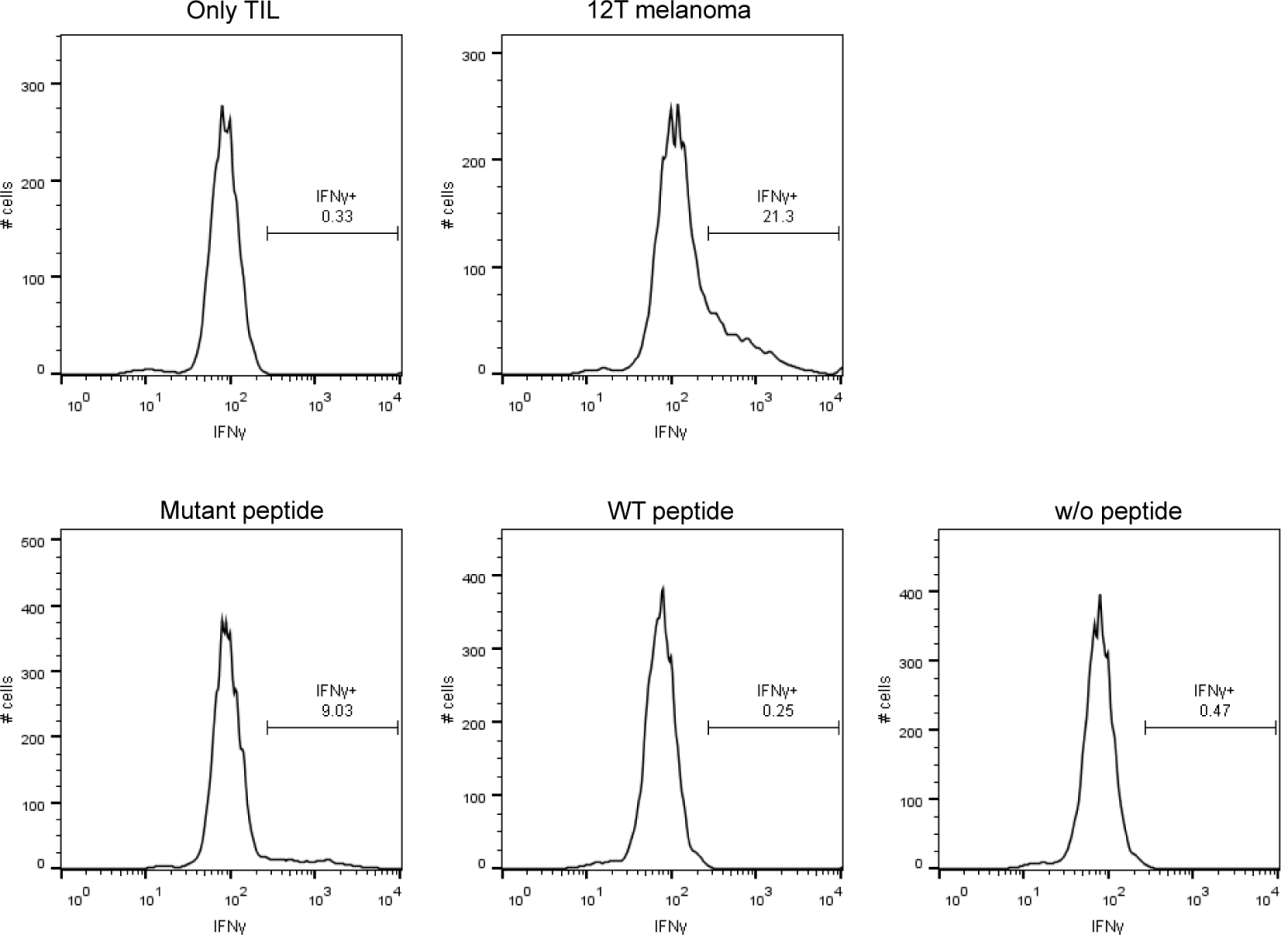
Intracellular staining of IFN-γ after stimulation with the peptides and melanoma Intracellular IFN-γ was stained after 6 hours co-culture of the TILs with T2 cells that were pulsed with 1μM of the mutated or wild-type peptides or with the autologous melanoma.

To further evaluate TILs reactivity with the mutant MED15 peptide, we preformed intracellular IFN-γ staining after co-culturing the TILs with the autologous melanoma as a reference for total reactivity or with T2 cells pulsed with the mutant or wild-type MED15 peptides. About 21% of the TILs were reactive against the melanoma cells and about 9% were reactive against the T2 cells pulsed with the mutant MED15 peptide. Therefore, we estimate that approximately 40% of the reactive TILs specifically react with the mutant peptide (Figure 4), suggesting that it is an immunodominant epitope.

### Prediction of neo-antigens using NetMHCpan algorithm

We were interested to compare our method to the currently prevalent algorithmic prediction method. To that end, we used The NetMHCpan 2.8 algorithm, to predict all possible neo-antigens derived from 12T non-synonymous mutations, for all the cells' HLA alleles and between 8-12 amino acids in length. In total, 5,404 different peptides were predicted (Supplementary Table 3).

Binding affinity predictions for wild-type and mutant peptides of MED15 and TPD52L2 are in Supplementary Table 4. The mutant and wild-type TPD52L2 peptides were predicted to bind the A*03 allele strongly and weakly, respectively and the MED15 mutant and wild-type peptide were predicted to weakly bind the B*51 allele.

## DISCUSSION

This report is the first to show that a combination of HLA peptidomics and autologous cancer exome data can be used to reveal cancer-specific antigens which expose neo-antigen, leading to a specific T cell response in humans. Currently, most neo-antigen identification studies use algorithms, such as NetMHCpan [4], to predict which mutated peptide sequences can bind the patients' HLA alleles. As the HLA peptide processing steps are not fully known, and therefore cannot be taken into consideration within the prediction algorithm, these predictions yield a large number of false positive hits, that must then all be screened. Several criteria are usually used to reduce the number of screened peptides. These include: predicted binding affinity, length of the predicted peptides and their expression levels. Applying such limitations can exclude peptides such as the mutated MED15 peptide which was predicted to weakly bind the B*51 molecule. In contrast, isolation of HLA peptides directly from tumor cells ensures that only peptides that are actually processed in the cells, bind the HLA and are presented on the cells' surface are tested for immunogenicity. Therefore more concerning than the added labor entailed by the multitude of candidate peptides, is the fact that the currently utilized filtering criteria also run the risk of producing false negative hits, thus missing peptides that might actually bind the HLA, as in the case demonstrated in this work.

The proposed method, depicted in Supplementary Figure 3, allows for identification of neo-antigens in significantly less time than currently used screening methods. While other methods require whole exome sequencing and identification of somatic mutations prior to wide-scale-screen with synthetic peptides, tetramers or construction of minigenes, mass spectrometry analysis of the peptides isolated from the tumor can be done in parallel, saving valuable time. Subsequent immunological assays for validation of the peptides identified by mass spectrometry only require synthesis of a few peptides compared to the hundreds of peptides that are needed for screening predicted sequences. Importantly, the number of reactive neo-antigens identified using this method is similar to the number of peptides identified by the other currently used methods (between 0-3 peptides per sample) [12, 13, 14, 6, 15].

As in all mass spectrometry analyses, identification is biased toward the most abundant peptides. Therefore, identifying a larger number of total HLA peptides will increase the chance of discovering neo-antigens. In this study HLA peptides were isolated from 2*10^8^ cells, which is the estimated number of cells in clinically detectable solid human tumors [16], making this method applicable for neo-antigen identification directly from the isolated tumor, without the need to establish a tumor cell line.

Finally, using flow cytometry analysis of intracellular IFNγ, we demonstrated that about 40% of TILs reactivity against the melanoma cells is attributable to specific reactivity to the mutated MED15 peptide. This result corroborates previous observations showing that autologous T- cell response is driven mainly by mutated neo-antigens [17, 18].

Here we provide, to our knowledge, the first demonstration of an approach that combines two powerful tools, forming an alternative system for identifying immunogenic neo-epitopes in human cancer. This approach may be used clinically as a potential diagnostic strategy for personalized immunotherapy, not only for melanoma patients but for a variety of additional tumor types where neo-antigens play a critical role in anti-tumor reactivity.

## MATERIALS AND METHODS

### Tumor tissues

All DNA samples used in this study were derived from metastases. Samples used for whole-exome capture were extracted from cell lines established directly from patient tumors as described previously [19]. We identified somatic mutations by comparing the tumor data to the matched normal tissue and identified 2,997 potential somatic mutations in 2,192 different genes. Of these alterations, 1019 caused amino acid changes consistent with prior data [19].

12T cells express the HLA-A*02, A*03, B*08, B*51, C*01 and C*07 class I alleles.

### Production and purification of membrane HLA molecules

Cells lysate from two pellets of 12T cell lines were used for immunoaffinity purification of HLA molecules with their bound peptides, using the W6/32 mAb, bound to Amino-Link beads (Thermo Scientific, as in [20, 21]). The HLA peptides were recovered from HLA molecules with 1% TFA followed by separation of the peptides from the proteins contaminants by binding the eluted fraction to disposable reversed-phase C18 columns (Harvard Apparatus). Elution of the peptides was done with 30% acetonitrile and 1% TFA. The eluted peptides were cleaned also by C18 stage tip as in [22].

### Identification of the HLA peptides

The HLA peptides were dried by vacuum centrifugation, re-solubilized with 0.1% Formic acid and resolved on capillary reversed phase chromatography on 0.075×200 mm laser-pulled capillaries, self-packed with 3μ Reprosil-Aqua C_18_ [23]. Electrospray tandem mass spectrometry was performed with the Q-Exactive-Plus mass spectrometer (Thermo Scientific). The MS data was analyzed by MaxQuant [10] version 1.4.1.2, with 5% FDR and by Sequest using the Proteome Discoverer version 1.4.1.14 (Thermo Scientific). Peptide identifications were based on the human section of the Uniprot database (http://www.uniprot.org) from February 2014 combined with the wild type and mutant protein sequences.

### Analysis of T cell responses

TILs were isolated and expanded from fresh tumor digests as previously described [24] and EBV-transformed B-cell lines were established from PBLs of the patient (EBV was a gift from Prof. Ephraim Gazit). The reactivity of the TILs were evaluated by incubating patient derived EBV transformed B cells or T2 cells (with antigen-processing defects that allow for the efficient loading of exogenous peptides [24]) with the candidate peptides at a concentration of 1 μM for 2 h at 37°C. Following three washing steps, TILs were added at a ratio of 1:1 (1e5 cells), and the amounts of soluble IFN-γ secreted from the TILs cultured overnight with peptide-pulsed target cells or tumor cells were measured by ELISA assay (Biolegend).

### Intracellular staining of IFN-γ

TILs were co-cultured with T2 cells pulsed with peptides or autologous melanoma cells for 6 hours. Then they were fixated using the Transcription Factor Buffer Set (BD Pharmingen), and stained anti IFN-γ, anti CD3 and anti CD8 (BD biosciences). Cells were analyzed using the FACSCalibur flow cytometer (BD biosciences). TILs were gated as positive for CD3 and CD8.

### cDNA sequencing

RNA was purified from cultured cells using RNAeasy mini kit (Qiagen) and cDNA was prepared using iScript reverse transcription supermix for RT-qPCR (Bio-Rad). The regions containing the mutation sites were amplified by PCR using KAPA HiFi HotStart ReadyMix PCR Kit (KAPAbiosystems). The PCR primer pairs used were: MED15 (5′- GGTGTCCCCTGAAGACCTTG) and (5′- CTGGGCCCAGGTGTTGAG); TPD52L2 (5′- GCCGGCCAAGATATCAACCT) and (5′- GATCCGACAGGGGCTTGTC). PCR was performed using 100 ng cDNA and the following parameters: 1 cycle at 95°C for 3 min; 30 cycles at 98°C for 20 sec, annealing temperature of 65°C for 15 sec, 72°C for 60 sec; and 1 cycle at 72°C for 1 min. PCR products were cleaned with QIAquick PCR Purification Kit (Qiagen) and sequenced using a 3730 DNA Analyzer (ABI). Sequencing primers were the same as the PCR primers.

### RNA sequencing

RNA was extracted from 12T cells using RNeasy mini kit (Qiagen). 1 μg of total RNA was processed using the TruSeq RNA Sample Preparation Kit v2 protocol (Illumina). Libraries were evaluated by Qubit and TapeStation. Sequencing libraries were constructed with barcodes to allow multiplexing of few samples on one lane of Illumina HiSeq 2500 V4 instrument. About 60 million single-end 60-bp reads were sequenced.

The TopHat (v2.0.10) was used to align the reads to the human genome (hg19), and counting reads on hg19 refseq genes (downloaded from igenomes) was done with HTSeq-count (version 0.6.1p1).

RPKM values were calculated using Total exonic length for each gene, which was calculated using the bioconductor GenomicFeatures package.

### Prediction of neo-antigens using NetMHCpan 2.8 algorithm

The residues surrounding the amino acids resulting from nonsynonymous mutations were scanned to identify candidate 8-12 mer peptides that were predicted to bind with high affinity (strong binders, %Rank≤0.5 or IC50≤50) or low affinity (weak binders, 0.5≤%Rank≤2 or 50≤IC50≤500) to the cells' HLA-I alleles using the NetMHCPan 2.8 algorithm [4].

## SUPPLEMENTARY MATERIAL FIGURES AND TABLES










