# Supplementary material for: Use of HLA peptidomics and whole exome sequencing to identify human immunogenic neo-antigens
Source: Oncotarget. 2016 Jan 20;7(5):5110–7. doi: 10.18632/oncotarget.6960 (PMC4868674; doi:10.18632/oncotarget.6960)
Supplement: Supplementary file 1 [file oncotarget-07-5110-s001.pdf]

Use of HLA peptidomics and whole exome sequencing to identify human immunogenic neo-antigens

Supplementary Material

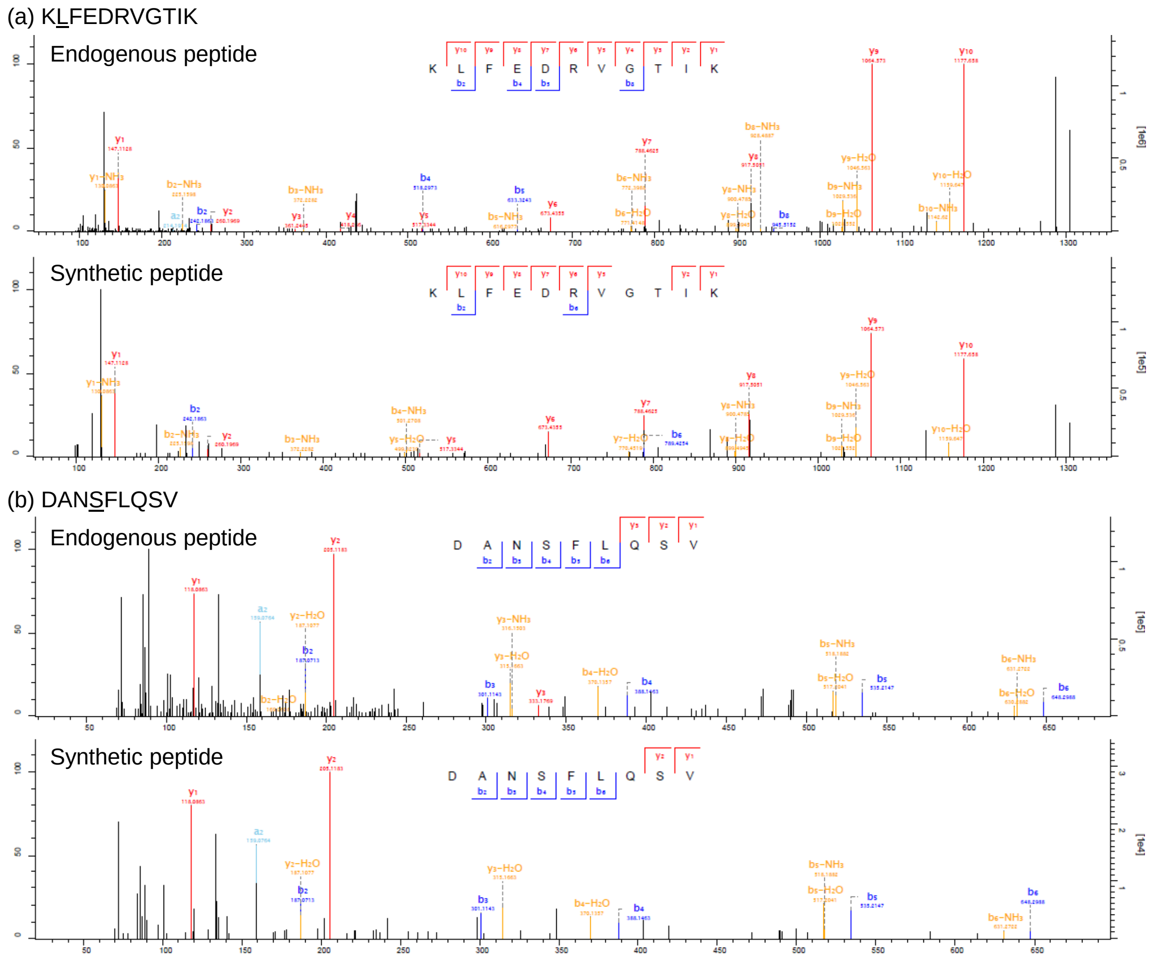

Supplementary Figure 1. Tandem mass spectra of endogenous mutant peptides identified in 12T and their corresponding synthetic peptides. A. KLFEDRVGTIK B. DANSFLQSV

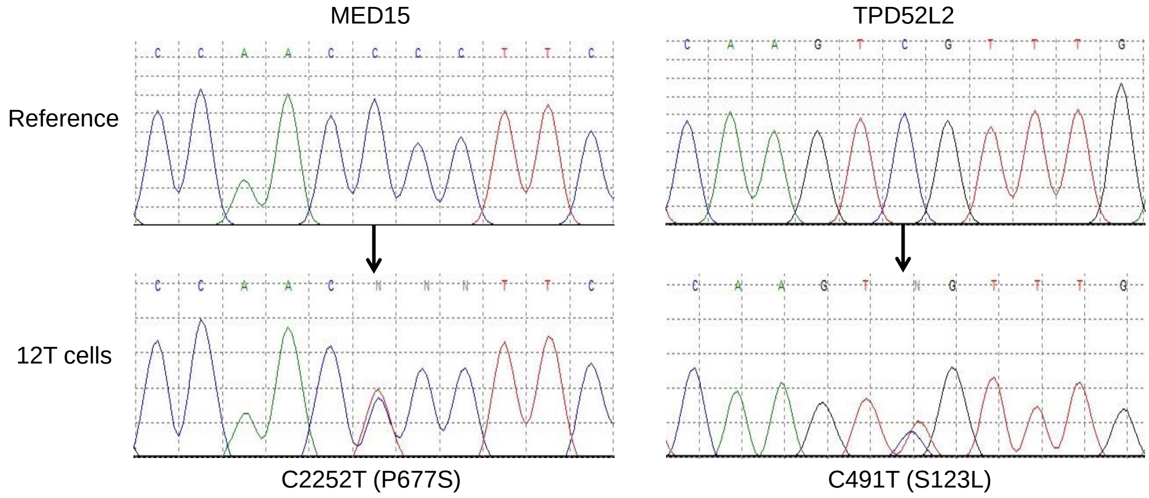

Supplementary Figure 2. Detection of mutations in MED15 and TPD52L2. Sanger sequencing of MED15 and TPD52L2 using cDNA from 12T cultured cells confirms the expression of mutant alleles. (a) MED15 (b) TPD52L2. In each case, the top sequence chromatogram is the reference sequence and the lower sequence chromatogram from the indicated tumors. Arrows indicate the location of missense mutations. The nucleotide and amino acid alterations are indicated below the chromatograms.

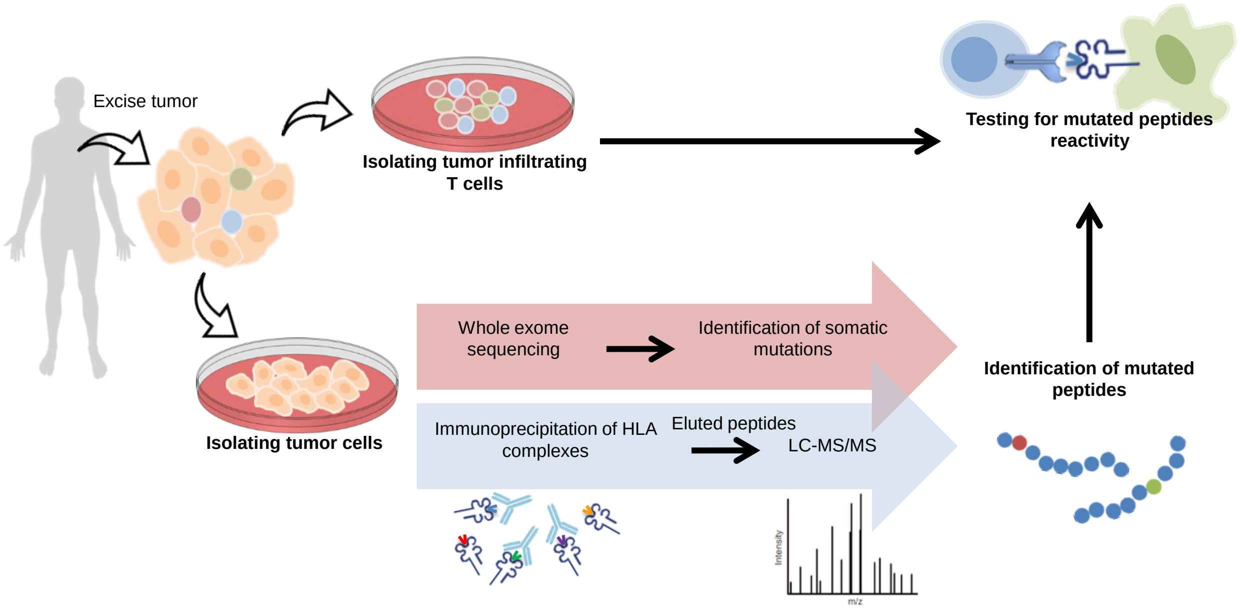

Supplementary Figure 3. Exon sequencing analysis combination with HLA peptidomics to Identify Human Immunogenic Neo-antigens. Schematic overview of the approach to identify HLA-I presented neo-antigens using a combination of whole exome sequencing and mass spectroscopy. Peptides were eluted from tumor HLA and analyzed against a customized peptide database which was based on the tumor whole exome sequencing. Immunogenicity of identified mutated peptides was validated using patient tumor infiltrating lymphocyte reactivity.
